# Effect of preparation design on the fracture behavior of 3D-printed restorations in an in-vitro tooth wear model

**DOI:** 10.1186/s12903-026-08031-0

**Published:** 2026-03-12

**Authors:** Thao Ly Nguyen-Thi, Thien Man Tran, Huyen Phuong Tran-Thi, Phuong Mai Nguyen-Ho, Thanh Tin Do, Minh-Huy Dang

**Affiliations:** 1 Faculty of Odonto-Stomatology, Hue University of Medicine and Pharmacy, Hue University, Hue City, Vietnam; 2 Center of Odonto-Stomatology, Hue Central Hospital, Hue City, Vietnam; 3 Faculty of Dentistry, Duy Tan University, Da Nang City, Vietnam

**Keywords:** 3D printing, Tooth wear, Occlusal veneer, Preparation design, Fracture resistance, Failure mode

## Abstract

**Background:**

Additive manufacturing technologies have introduced new nanohybrid resin composites for permanent indirect restorations. However, limited evidence exists regarding the mechanical performance of 3D-printed restorations in managing occlusal wear. This study evaluated the effect of preparation design on fracture resistance, preparation time, and failure modes of 3D-printed restorations.

**Methods:**

Forty maxillary premolars were allocated to four groups (*n* = 10): sound teeth (ST), overlay (OV), veneerlay (VL), and full crown (FC). Experimental groups received standardized 1.0–1.2 mm occlusal wear simulation. Restorations were designed using CAD software and fabricated with a 3D-printed nanohybrid composite (Rodin Sculpture 2.0). Cementation employed immediate dentin sealing and heated composite resin. Specimens underwent compressive fracture testing in a universal testing machine. Failure modes were categorized as catastrophic and non-catastrophic. One-way ANOVA with Tukey’s post-hoc test and Fisher’s exact test were used (α = 0.05).

**Results:**

Preparation design significantly influenced fracture resistance (*p* < 0.05). OV exhibited the highest mean fracture load (1842.82 ± 560.47 N), which was significantly higher than FC (1236.79 ± 497.59 N). Preparation time increased with the invasiveness of the design (OV < VL < FC; *p* < 0.05). Catastrophic failures occurred most frequently in the FC group (60%) and were not observed in ST or OV.

**Conclusions:**

3D-printed occlusal veneers demonstrated fracture resistance values that exceeded physiological loading, with overlays providing superior strength and the lowest risk of catastrophic failure. Conservative adhesive preparation designs should be favored over full crowns in the rehabilitation of worn dentition.

**Supplementary Information:**

The online version contains supplementary material available at 10.1186/s12903-026-08031-0.

## Background

Tooth wear has increasingly emerged as a significant clinical and public health concern, particularly among younger populations, reflecting changes in lifestyle, diet, and parafunctional habits [1]. According to the FDI World Dental Federation (2023), it is defined as the cumulative loss of mineralized tooth structure caused by physical or chemophysical processes unrelated to dental caries [2]. This progressive loss can compromise oral function and aesthetics, leading to hypersensitivity, pulpitis, loss of vertical dimension of occlusion, and temporomandibular disorders [1, 3]. Once pathological tooth wear is diagnosed, timely intervention is recommended, with current guidelines emphasizing additive and conservative strategies that preserve sound, healthy tooth structure [4, 5]. Within this framework, occlusal veneers have emerged as a key minimally invasive restorative option, offering a biologically favorable alternative to conventional full-coverage crowns in the rehabilitation of worn dentitions [5].

Longevity of restorations is strongly influenced by both mechanical and biological factors, with fracture resistance playing a critical role [6, 7]. Although the clinical effectiveness of occlusal veneers fabricated from ceramic or hybrid materials has been well established, conventional CAD/CAM milling workflows present several limitations, including higher production costs, extended fabrication time, restricted capability in reproducing complex geometries, and limited options for intraoral repair [8, 9]. To overcome these constraints, three-dimensional (3D) printing technology has emerged as a promising alternative [8, 9]. Early evidence supports its feasibility in the production of occlusal veneers and highlights its substantial potential within restorative dentistry broadly, and in the management of tooth wear specifically [10, 11].

CAD/CAM technology enables the fabrication of thin and ultrathin occlusal veneers, with thicknesses ranging from 0.3 to 1.5 mm, depending on the materials and permits a variety of preparation designs that can be tailored to specific clinical situations to restore the occlusal function and morphology of tooth-wear defects [12, 13]. These designs—comprising tabletops (covering all cusps and restricted to the occlusal surface), overlays (extending to the proximal and free surfaces with a circumferential chamfer that increases the prepared area), and veneerlays (indicated in cases of esthetic impairment of the buccal surface)—have a significant impact on the fracture behavior of restored teeth [12].

Advancements in adhesive and material technology have positioned minimally invasive restorative solutions, such as occlusal veneers, as increasingly viable alternatives to traditional full-coverage crowns in the rehabilitation of tooth wear [10, 11]. Considering emerging trends, including same-day dentistry, in-house dentistry, and fully digital workflows—each receiving significant attention—coupled with the alarming rise in tooth wear among younger populations, there is a clear need for further research to explore the potential of 3D printing technology in managing pathological tooth wear.

The aim of this study was to evaluate the impact of preparation design on fracture resistance, preparation time, and failure modes of maxillary premolars, simulated with artificial tooth wear and restored using 3D-printed materials. The null hypothesis posited that there was no significant difference in fracture resistance or failure modes among the different preparation designs.

## Methods

### Specimen preparation

Forty human maxillary premolars extracted for orthodontic purposes from patients aged 18–25 were collected. Teeth presenting caries, restorations, visible cracks, or abnormal morphology were excluded. Only teeth with normal anatomical morphology, one or two roots, and similar crown dimension were included. Ethical approval for the use of human teeth was obtained from the Ethics Committee of Hue University of Medicine and Pharmacy, Hue University, Hue City, Vietnam (Approval No. H2024/124). Informed consent was obtained from all donors in accordance with the Declaration of Helsinki. The study was carried out between March 2024 and July 2025. Laboratory procedures were performed at the Faculty of Odonto-Stomatology, Hue University of Medicine and Pharmacy, Hue University (Hue City), while mechanical testing was conducted at the Quality Assurance and Testing Center 3 (Da Nang City), Vietnam.

**Fig. 1:**
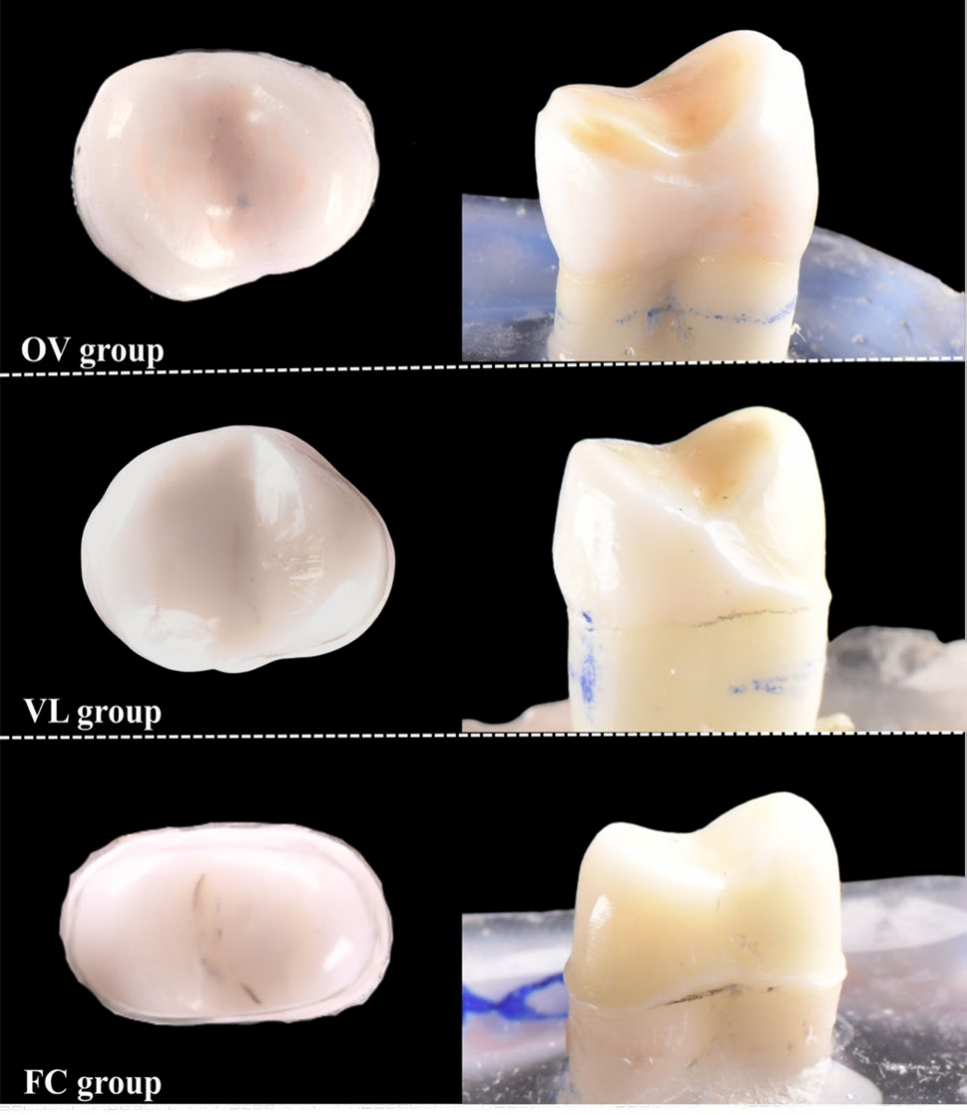
Preparation designs

All specimens were thoroughly cleaned to remove external debris and subsequently stored in T-chloramine solution for one month. Following storage, each tooth was embedded in an acrylic resin block, leaving 2–3 mm of root surface exposed apical to the cementoenamel junction (CEJ). Ten of the forty teeth were randomly assigned to the control group (sound teeth). The remaining thirty teeth underwent standardized occlusal reduction of 1.0–1.2 mm to simulate tooth wear. The detailed step-by-step protocol for occlusal reduction is illustrated in Supplementary Figure S1. After occlusal reduction, these specimens were randomly distributed into three experimental groups according to preparation design: Overlay, Veneerlay, Full crown (Fig. 1). The sample size for each group (*n* = 10) was determined in accordance with the recommendations for in vitro testing of polymer-based materials as specified in ISO 4049. This sample size is also consistent with established methodologies used in recent comparable studies by Martins et al. (2023) and Pranit et al. (2024) [12, 14].

A single operator performed all operative phases, including specimen preparation, dentin sealing, and cementation. The preparation time for each specimen was recorded, encompassing both the tooth preparation and dentin sealing phases.

### Tooth preparation

All teeth were prepared using a red-ring high-speed handpiece (Foshan COXO Medical Instrument Co., Ltd., Foshan, China) and diamond burs (Komet Dental, Lemgo, Germany and Kerr, Brea, CA, USA) following the protocols described by W. F. Martins et al. and D. Edelhoff et al. [12, 15]. The detailed step-by-step protocol for tooth preparation is illustrated in Supplementary Figures S2, 3, and 4.

### Immediate Dentin Sealing (IDS)

To enhance bond strength, an immediate dentin sealing (IDS) procedure was performed. The exposed dentin was air-dried, and a universal adhesive (Single Bond Universal, 3M ESPE, St. Paul, MN, USA) was applied for 15 s, gently air-thinned, and light-cured for 20 s using an LED unit (Elipar™ DeepCure, 3M ESPE, St. Paul, MN, USA). A thin layer of flowable composite (Filtek™ Z350 XT Flowable, shade A3, 3M ESPE, St. Paul, MN, USA) was then applied, evenly spread over the sealed dentin, and light-cured for 20 s. Glycerin gel was used to block the oxygen-inhibited layer, followed by an additional 40 s of light-curing (Elipar™ DeepCure, 3M ESPE, St. Paul, MN, USA). Excess material was carefully removed, and the tooth surfaces were finished and polished using Sof-Lex discs (3M ESPE, St. Paul, MN, USA) and Eve Diacomp Twist (EVE Ernst Vetter GmbH, Keltern, Germany).

### Design of restorations and manufacturing

**Fig. 2:**
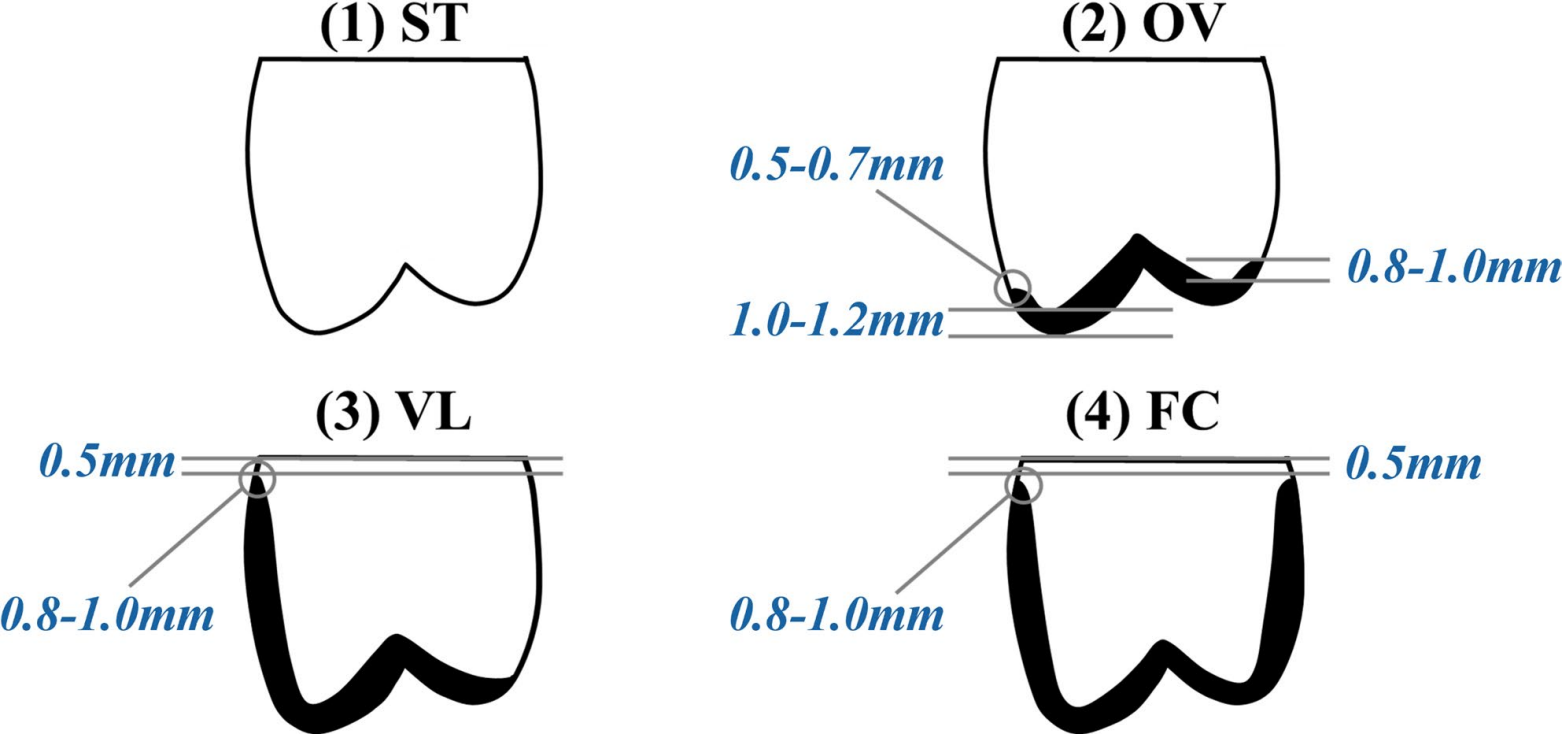
(1) ST group: Sound Tooth (Control group); (2) OV group: 1.0–1.2 mm occlusal reduction featuring a 0.5–0.7 mm chamfer finish line prepared within the enamel; (3) VL group: 1.0–1.2 mm occlusal reduction combined with buccal preparation and a 0.8–1.0 mm chamfer finish line; (4) FC group: 1.0–1.2 mm occlusal reduction and circumferential axial preparation with a 0.8–1.0 mm chamfer finish line.

Digital impressions of the prepared teeth were obtained using an intraoral scanner (Medit i700 and Medit Link, Medit, Seoul, Korea). The restorations were designed in 3Shape TRIOS Design Studio (3Shape, Copenhagen, Denmark) based on the maxillary premolar morphology. Each restoration was designed with an occlusal thickness of 1.0–1.2 mm and a cement space of 0.02 mm. Before manufacturing, all CAD files were reviewed and adjusted to ensure conformity with the required dimensions, morphology, and margin quality (according to Fig. 2). The restoration design and positioning of support tips are illustrated in Supplementary Figure S8.

The restorations were printed using Rodin Sculpture 2.0 nanohybrid composite (Rodin Sculpture 2.0; shade A1; zirconia-infused nanohybrid resin, > 60% filler content; Pac-Dent, Brea, CA, USA) with a WhipMix VeriEKO 3D printer (WhipMix, Louisville, KY, USA). Post-printing procedures were performed according to the manufacturer’s instructions. Based on the manufacturer’s technical data, Rodin 2.0 is a ceramic-filled (zirconia-containing) printable nanohybrid resin with a reported filler content of approximately 60% (w/w), reported biaxial flexural strength > 200 MPa, and radiopacity.

All CAD/CAM procedures, including design and printing, were carried out at Vvtech Lab in Hanoi city, Vietnam. Prior to cementation, necessary adjustments were made to ensure proper fit. Any restoration that did not meet the criteria for morphology, dimensions, or marginal adaptation was reprinted.

### Adhesive cementation

#### Tooth conditioning

The enamel and previously treated IDS surfaces were subjected to airborne-particle abrasion using 29-µm aluminum oxide powder for 10 s (AquaCare TWIN, Velopex, London, UK, at 5 bar pressure, perpendicular, and at a distance of 10–15 mm). Following this, the surfaces were etched with 35% phosphoric acid (Prime Dent, Chicago, IL, USA) for 30 s, rinsed for 15 s, and air-dried. A thin layer of universal adhesive (Single Bond Universal, 3M ESPE, St. Paul, MN, USA) was then applied for 20 s, followed by air-thinning without light curing. The detailed step-by-step protocol is illustrated in Supplementary Figure S5a, b.

#### Restoration conditioning

The intaglio surfaces of the 3D-printed restorations were subjected to airborne-particle abrasion using 29-µm aluminum oxide powder for 10 s (AquaCare TWIN, Velopex, London, UK, at 5 bar pressure, perpendicular, and at a distance of 10–15 mm). After abrasion, the restorations were immersed in distilled water in an ultrasonic bath for 4 min. Silane (Monobond-S, Ivoclar Vivadent) was then applied for 60 s, air-dried, and a thin layer of universal adhesive (Single Bond Universal, 3M ESPE, St. Paul, MN, USA) was applied for 20 s, followed by air-thinning without light curing. The detailed step-by-step protocol is illustrated in Supplementary Figure S5a, b.

#### Cementation

A heated composite resin (Filtek Z250, shade A1, 3M ESPE, St. Paul, MN, USA , at 60 °C) was placed on the internal surface of the restoration, which was then seated onto the tooth. Excess material was removed, and polymerization was performed using an LED curing unit (Elipar™ DeepCure, 3 M ESPE) in three cycles of 20 s each, applied sequentially from the occlusal, buccal, and palatal aspects. Glycerin gel was applied to block the oxygen-inhibited layer, followed by an additional 40 s of light curing on each side. Final finishing and polishing of the margins were performed using Sof-Lex discs (3M ESPE, St. Paul, MN, USA) and the Eve Diacomp Twist (EVE Ernst Vetter GmbH, Keltern, Germany). The detailed step-by-step protocol is illustrated in Supplementary Figure S5c, d.

All specimens were stored in distilled water at room temperature for 7 days before further testing. Supplementary Figure S6 shows the three restored groups after finishing and polishing.

#### Fracture resistance testing

All 40 specimens were tested for fracture resistance using a universal testing machine (Shimadzu UH-500kNXh, Shimadzu Corp., Kyoto, Japan). A load was applied parallel to the long axis of the tooth using a stainless-steel indenter, featuring a 4 mm diameter with a blunt (rounded) tip at a crosshead speed of 0.5 mm/min. The indenter was positioned to simultaneously contact both the buccal and lingual cusps surrounding the central fossa of the restoration. A 0.2-mm-thick layer of tin foil was placed between the restorations and the indenter to minimize stress concentration and simulate the presence of a food layer. The specimens were subjected to compressive load in a distilled water bath at room temperature until fracture occurred. The maximum load to fracture was recorded as the fracture resistance, measured in Newtons (N). The experimental set-up for the in vitro compressive load test is illustrated in Supplementary Figure S7.

### Failure analysis

The failure of the specimens was examined visually and through digital photography to identify evidence of cracks, adhesive failures, or cohesive failures. The failure modes of each specimen were classified according to the following system [16, 17]:

1. Extensive crack formation within the restoration (on natural enamel for sound tooth.
2. Cohesive fracture within the restoration or mixed failure (cohesive and adhesive) without dental involvement (the enamel and dentin of crown involved for sound tooth.
3. Fracture within the restoration and tooth crown structure.
4. Longitudinal restoration and tooth fracture involving the root.

Mode 1, 2, and 3 were non-catastrophic fractures and mode 4 was catastrophic fracture.

**Fig. 3:**
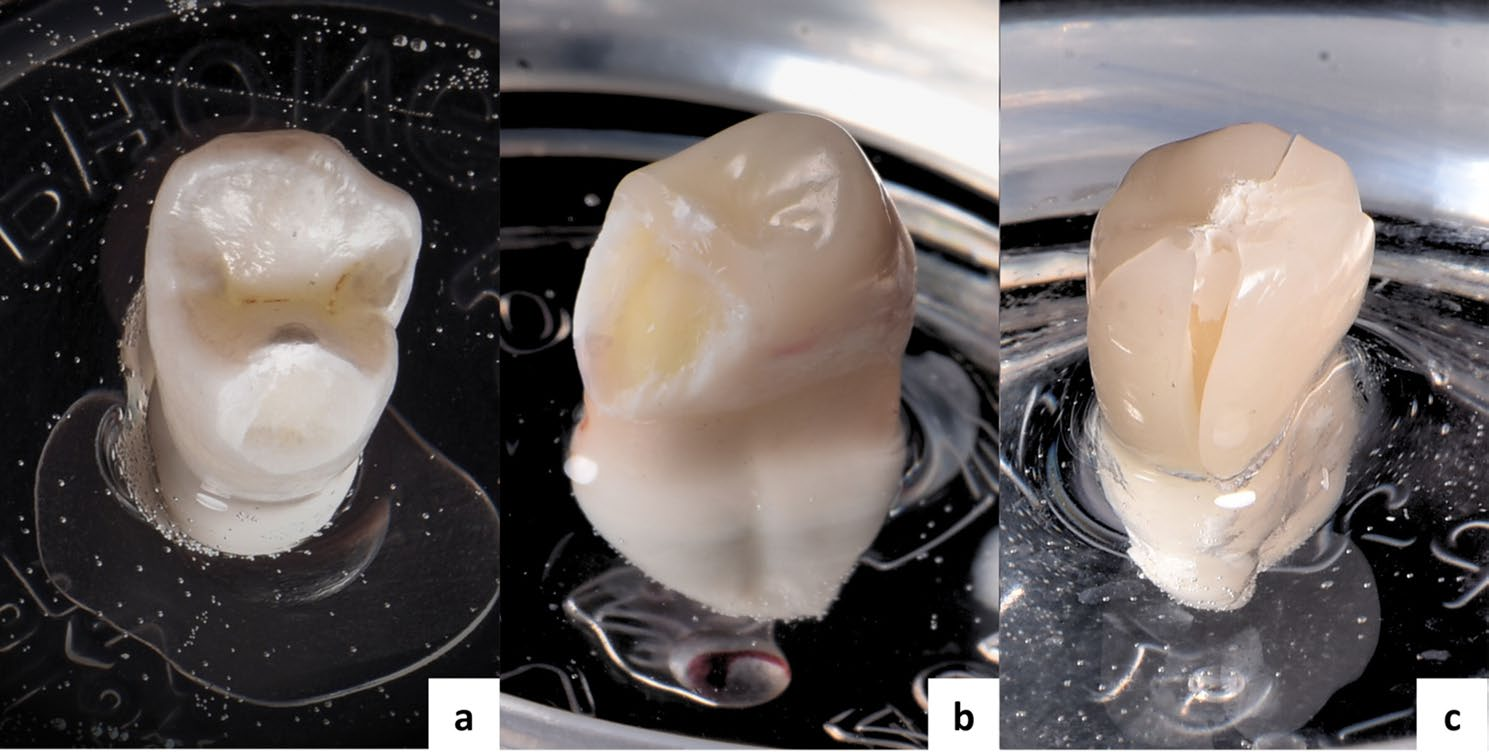
Representative failure modes observed in the study. (**a**) Mode 1 in the sound teeth (ST) group; (**b**) Mode 3 in the restored groups; (**c**) Mode 4 in the restored groups

Figure 3 shows representative failure modes observed in the study.

### Statistical analysis

Statistical analysis was performed using Microsoft Excel 2016 (Microsoft Corp., Redmond, WA, USA) and RStudio with R 4.3.0 (Posit Software, PBC, Boston, MA, USA). Fracture resistance and preparation time data were analyzed using one-way ANOVA, followed by Tukey’s post-hoc test. Failure mode data were evaluated using Fisher’s exact test. The level of statistical significance was set at α = 0.05.

## Results

### Fracture resistance

**Fig. 4:**
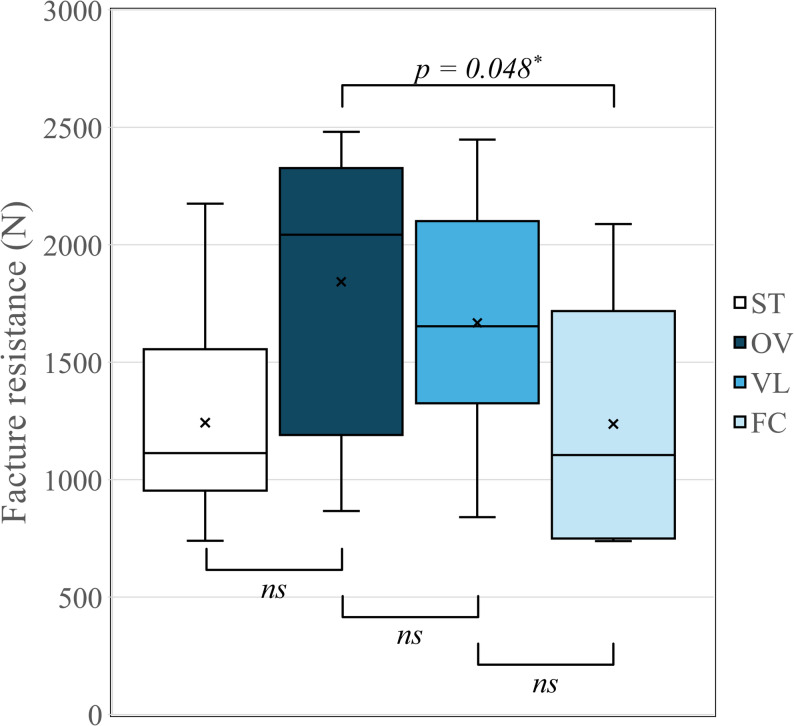
Boxplot graph representing the mean fracture resistance (N) of the groups. Data were analyzed using one-way ANOVA and Tukey’s post-hoc test. Asterisks indicate statistically significant differences (*: p <0.05). ns: not significant

The mean fracture resistance (N) and standard deviation of four groups are shown in Table 1 and illustrated in Fig. 4.

One-way ANOVA test showed that there were significant differences among the groups (*p* = 0.019). Tukey’s post-hoc test demonstrated that the OV group had a significantly higher fracture load than the FC group (*p* = 0.048). The ST, VL, and FC showed no statistically significant differences.

**Table 1:** Mean fracture resistance (*N*) and standard deviation of four groups

Group	Mean	Standard deviation	Minimum - Maximum
ST (*n* = 10)	1242.66	438.25	739.18–2174.93
OV (*n* = 10)	1842.82	560.47	866.33–2481.06
VL (*n* = 10)	1667.36	497.54	840.5–2447.45
FC (*n* = 10)	1236.79	497.59	738.15–2088.87

### Preparation time

**Fig. 5:**
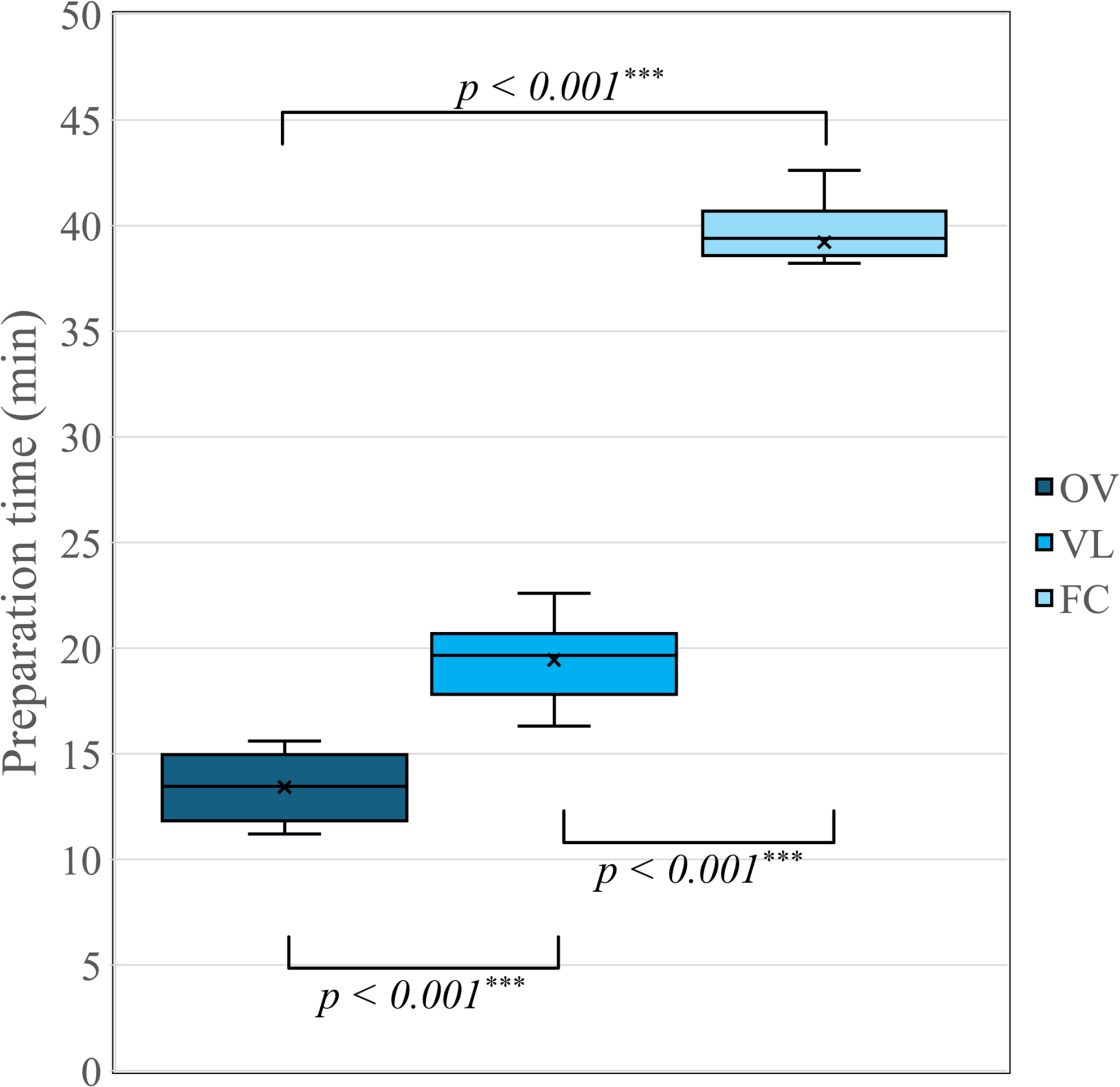
Boxplot graph representing the mean preparation time (min) of the groups. Data were analyzed using one-way ANOVA and Tukey’s post-hoc test. Asterisks indicate statistically significant differences (*: p <0.05, ***: p <0.001). ns: not significant

The mean preparation time (min) and standard deviation of four groups are shown in Table 2 and illustrated in Fig. 5.

One-way ANOVA revealed significant differences among the groups (*p* < 0.001). Tukey’s post-hoc test further indicated significant differences between the groups, with preparation time increasing in the following order: OV < VL < FC (*p* < 0.001).

**Table 2:** Mean preparation time (min) and standard deviation of four groups

Group	Mean	Standard deviation	Minimum - Maximum
OV (*n* = 10)	13.42	1.69	11.20–15.60
VL (*n* = 10)	19.44	1.92	16.30–22.60
FC (*n* = 10)	39.21	2.68	32.60–42.60

### Failure modes

**Fig. 6:**
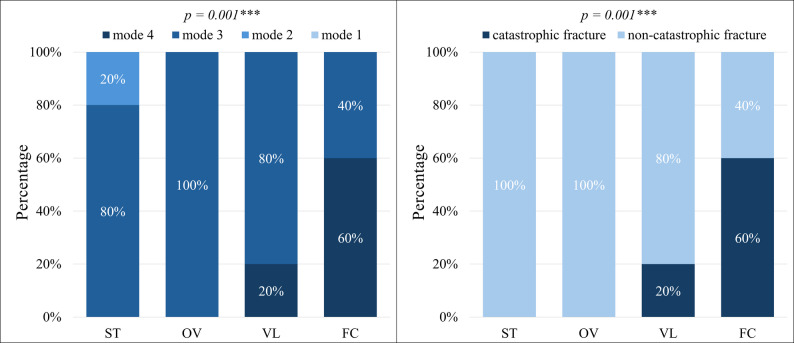
Bar charts depicting percentages of the failure modes of four groups. Data were analyzed using Fisher's exact test. Asterisks indicate statistically significant differences (***: p <0.001). ns: not significant

The failure mode analysis of the four groups after compressive loading is summarized in Table 3 and depicted in Fig. 6. Fisher’s exact test indicated significant differences among the groups (*p* = 0.001). Mode 1 failure was absent in all groups, while mode 3 failure was detected in all groups. Mode 4 failure was observed in the VL and FC groups.

The proportion of mode 4 failure—characterized by longitudinal restoration and tooth fracture involving the root—showed an increasing trend in the following order: ST and OV < VL < FC (*p* = 0.001). This catastrophic failure type was most prevalent in the FC group, with a frequency exceeding 50%.

**Table 3:** Distribution of the fracture modes of four groups

Group	mode 1	mode 2	mode 3	mode 4	catastrophic fracture	non-catastrophic fracture
ST (*n* = 10)	0 (0%)	2 (20%)	8 (80%)	0 (0%)	0 (0%)	10 (100%)
OV (*n* = 10)	0 (0%)	0 (0%)	10 (100%)	0 (0%)	0 (0%)	10 (100%)
VL (*n* = 10)	0 (0%)	0 (0%)	8 (80%)	2 (20%)	2 (20%)	8 (80%)
FC (*n* = 10)	0 (0%)	0 (0%)	4 (40%)	6 (60%)	6 (60%)	4 (40%)

## Discussion

Fracture resistance is a fundamental prerequisite for the biomechanical stability and clinical longevity of restored teeth under physiological masticatory forces [18, 19]. In line with current minimally invasive restorative concepts, preparation designs should preserve sound tooth structure as much as possible while maintaining sufficient material thickness to achieve the necessary mechanical strength.

In the premolar region, physiological biting forces typically range from 222 to 445 N, with peak loads potentially reaching 800 N during clenching. Within the parameters of this in-vitro study, the groups restored with 3D-printed nanohybrid composites exhibited mean fracture resistance values between 1236 and 1842 N, which are substantially higher than the maximum reported physiological occlusal loads [16, 20]. Notably, the fracture resistance of all three experimental groups was found to be comparable to that of the sound teeth (control) group, with no statistically significant differences observed. These results suggest that the tested materials and designs may offer the necessary initial strength to meet functional clinical demands. This favorable mechanical behavior is likely attributed to the high ceramic filler content in the composition of Rodin Sculpture 2.0 (up to 60% according to the manufacturer), which contributes to its enhanced structural durability and resistance to static loading [21]. Collectively, these findings provide preliminary evidence of the potential of 3D-printed materials for definitive restorations, particularly when utilizing occlusal veneers with a thickness of 1.0–1.2 mm in the rehabilitation of worn dentition.

The null hypothesis was rejected, as significant differences were observed in fracture resistance as well as in the distribution of failure modes between the different preparation designs.

In terms of fracture resistance, the results of this study suggest that minimally invasive occlusal veneers, specifically overlays, demonstrate enhanced biomechanical performance compared to traditional full-coverage crowns when restoring occlusal wear. A clear correlation was observed where fracture resistance progressively decreased as more sound tooth structure was sacrificed.

These findings align with previous investigations by Ferraris et al. and X. Huang et al. on lithium disilicate restorations, which similarly highlighted the superior fracture resistance of minimally invasive techniques over full crowns [16, 17]. Ferraris et al. observed that a full bevel design yielded higher fracture resistance than butt joint, shoulder, or full crown configurations [17]. This performance was attributed to two critical factors: (1) the preservation of proximal ridges, and (2) the optimization of the enamel bonding surface [17]. Similarly, X. Huang et al. demonstrated an inverse correlation between axial reduction and fracture resistance, ranking resistance in descending order from Type O (no axial reduction) to Full Crowns (four-wall reduction) [16]. These results underscore the vital importance of preserving load-bearing components, such as axial walls and marginal ridges, alongside an optimized bonding interface to enhance the overall durability of the restoration.

The clinical advantage of minimally invasive approaches is further supported by their ability to preserve up to 40% more tooth structure than traditional crowns [22]. Given that restorative materials generally exhibit lower resistance to oblique forces compared to vertical axial forces, the extensive removal of healthy axial walls to achieve a full crown morphology may be counterproductive in the rehabilitation of occlusal wear [23]. In the present study, the overlay design—by retaining greater structural integrity—exhibited higher fracture resistance. Furthermore, since the full crown design relies heavily on an adhesive mechanism, the extensive removal of enamel at the finishing margin likely compromised both bond strength and the ultimate resistance to fracture.

Our data also indicated that the overlay design achieved higher fracture resistance than the veneerlay, although the difference did not reach statistical significance. This trend is consistent with findings by W. F. Martins et al. regarding semi-direct composite veneers and the Finite Element Analysis (FEA) performed by X. Huang et al. [12, 24]. Therefore, in the rehabilitation of worn dentition, minimally invasive treatments should be considered a priority. While the overlay design appears to offer the highest mechanical resistance in this experimental setup, the veneerlay remains a viable alternative in cases with higher aesthetic demands, such as those requiring modifications to the tooth’s external morphology or color.

The data from this study suggest that preparation design significantly influences the failure characteristics of teeth restored with 3D-printed nanocomposites, with an increasing incidence of root-related (catastrophic) failures as more healthy tooth structure is sacrificed. The occlusal veneer groups, particularly the overlays, demonstrated a lower risk of root-related failures compared to the full crown group. Of particular interest, when failure occurred, the overlay group exhibited a favorable repairability pattern consistent with that of the sound teeth (control) group. This observation suggests a potential biomechanical advantage of the overlay design; by prioritizing the preservation of sound tooth structure, it appears to favor failure behaviors that are more aligned with those of an intact tooth, potentially facilitating repair rather than catastrophic failure. This trend aligns with findings by W. F. Martins et al., who reported a higher risk of catastrophic fractures in veneerlay designs compared with overlays [12]. Furthermore, R. E. Campos et al. observed that the predominant failure mode in indirect composite crowns was related to the root structure, a pattern that mirrors our observations in the full crown group [25].

Both these results and our findings demonstrate the favorable repairability of occlusal veneers, particularly the overlay design, compared to traditional full crowns. The observed failure behaviors can be attributed to several interacting biomechanical factors: (1) the remaining healthy tooth structure and the bonding enamel surface; (2) the restorative material’s compatibility with the preparation design and adhesive system; and (3) the masticatory forces and the biomechanical conditions in the oral environment [25]. When axial forces (along the tooth axis) are applied, internal stress is generated within the restoration as a molecular response to external loading [26]. The distribution of this stress is highly dependent on the elastic modulus of the materials involved [27]. For materials with an elastic modulus similar to dentin, such as the 3D-printed nanohybrid composite used in this study (Rodin Sculpture 2.0, approximately 12 GPa) and the resin cement (Filtek Z250, approximately 18 GPa), stress tends to be distributed more uniformly and transferred effectively to adjacent layers [21, 27–29]. Given that optimal bond strength was achieved in this study, stress was likely transmitted through the cement layer to the underlying tooth structure without significant adhesive interface degradation. This is further supported by the clinical observation that no debonding failures (either cohesive or adhesive) were recorded in any specimen.

In preparation designs that extend axial walls toward the cervical area—specifically veneerlays with buccal extensions and full crowns with circumferential walls—a significant portion of shear stress develops and concentrates in the root region [27]. Due to the dentin-like elastic modulus of the restorative and luting materials, this shear stress propagates toward the cervical region, thereby increasing the risk of crack formation beneath the Cementoenamel Junction (CEJ) [21, 29]. The lower incidence of catastrophic failure in the veneerlay group compared to full crowns may be attributed to the smaller finish line area in proximity to the CEJ. Conversely, the favorable failure modes observed in the overlay group, which were primarily contained above the cervical region, are likely a result of preserving the axial tooth structure and utilizing a larger enamel bonding surface. This structural preservation helps prevent stress from propagating into the root region, significantly reducing the risk of irreversible tooth damage.

The findings of this study indicate that restoration design is a primary factor influencing clinical efficiency, particularly regarding preparation time. Within the parameters of our investigation, the time required for full crown preparation was found to be more than double that of the two occlusal veneer groups. This significant difference is likely attributed to the simplified preparation requirements of occlusal veneers, which eliminate the need for circumferential axial reduction. Furthermore, the supragingival finish lines characteristic of occlusal veneers provide a highly visible working field, minimizing marginal gingival trauma and facilitating the impression-taking process, whether traditional or digital [15]. Additionally, this design streamlines the cementation phase, as the accessible margins allow for more controlled removal of excess luting agent and simplified polishing procedures. Moreover, these supragingival margins facilitate more effective at-home oral hygiene for the patient, allowing for easier daily plaque control compared to subgingival alternatives [15]. The integration of 3D printing technology further enhances the efficiency of this conservative approach by enabling the simultaneous manufacturing of multiple restorations, which potentially optimizes laboratory turnaround time and production costs. As a result, the synergy between the efficient design of occlusal veneers and the capabilities of 3D printing offers a more streamlined alternative for the management of worn dentition. By not only reducing chairside time but also minimizing discomfort caused by dentin exposure during the preparation process, this combination significantly enhances the overall patient experience [30]. In cases where an increased vertical dimension of occlusion (VDO) provides sufficient space, these restorations can even be delivered via a non-prep approach, further emphasizing the time and cost-effectiveness of this digital strategy for tooth wear rehabilitation [30]. Despite the promising findings, certain limitations should be considered. First, regarding the tooth wear simulation model, the use of fresh dentin (achieved by reducing intact tooth structure) does not fully replicate the sclerotic dentin typically encountered in clinical cases of pathological wear. Sclerotic dentin possesses distinct structural and mineral characteristics that could significantly influence the long-term effectiveness of adhesive protocols and may require specific surface treatment methods to optimize adhesion quality. Second, the experimental setup utilized axial static loading, which represents the simplest mechanical scenario. This approach fails to account for oblique forces, cyclic fatigue, and thermomechanical aging. Furthermore, the absence of the periodontal complex—specifically the periodontal ligament and supporting bone—limits the accurate simulation of stress distribution and tooth mobility in a dynamic oral environment. Addressing these challenges in future research is essential to bridge the gap between in-vitro findings and clinical practice. Implementing advanced testing protocols—such as cyclic fatigue loading within a chewing simulator and incorporating Finite Element Analysis (FEA)—would allow for a deeper understanding of stress distribution throughout the entire tooth-restoration-periodontium complex. Ultimately, while these laboratory results provide a strong foundational baseline, long-term longitudinal clinical trials remain the gold standard required to fully validate the clinical performance and longevity of 3D-printed occlusal veneers.

## Conclusions

Within the limitations of this study, the following conclusions can be drawn:

1. Material Potential: Premolar teeth restored with 3D-printed nanohybrid composites at an occlusal thickness of 1.0 - 1.2 mm exhibit fracture resistance exceeding average human masticatory forces. These findings suggest the potential of this material for use in definitive posterior restorations, provided further clinical validation is conducted.
2. Influence of Design: Restoration design significantly affects biomechanical performance; as healthy tooth structure is progressively lost, fracture resistance tends to decrease while the risk of catastrophic failure increases. Among the evaluated designs, the occlusal overlay demonstrated favorable fracture resistance and failure modes, along with greater time efficiency compared to traditional full crowns within this experimental setup.
3. Clinical Relevance: In the rehabilitation of worn dentition, minimally invasive adhesive treatments, such as occlusal veneers, may offer a viable and conservative alternative to conventional full crowns, potentially optimizing both clinical chairside time and patient comfort within a digital workflow.

## Supplementary Information

Supplementary Material 1.

## Data Availability

All data generated or analyzed during this study are included in this published article and its supplementary information files. Additional raw data are available from the corresponding author on reasonable request.

## Abbreviations

3D: Three-dimensional
ST: Sound teeth
OV: Overlay
VL: Veneerlay
FC: Full crown
IDS: Immediate dentin sealing
CEJ: Cementoenamel junction
ANOVA: Analysis of variance
