# Supplementary material for: Effect of preparation design on the fracture behavior of 3D-printed restorations in an in-vitro tooth wear model
Source: BMC Oral Health. 2026 Mar 12;26:704. doi: 10.1186/s12903-026-08031-0 (PMC13094187; doi:10.1186/s12903-026-08031-0)
Supplement: Supplementary file 1 — Supplementary Material 1. [file 12903_2026_8031_MOESM1_ESM.docx]

**Effect of Preparation Design on the Fracture Behavior of 3D-Printed Restorations
in an In-Vitro Tooth Wear Model**

**Authors:** Thao Ly Nguyen-Thi¹**,** Thien Man Tran¹, Huyen Phuong Tran-Thi¹, Phuong Mai Nguyen-Ho², Thanh Tin Do³**,** Minh-Huy Dang ¹*

**Affiliations:**

¹ Faculty of Odonto- Stomatology, Hue University of Medicine and Pharmacy, Hue University, Hue City, Vietnam

² Center of Odonto- Stomatology, Hue Central Hospital, Hue City, Vietnam

³ Faculty of Dentistry, Duy Tan University, Da Nang City, Vietnam

**Corresponding author:**

Dr. Minh-Huy Dang

Faculty of Odonto- Stomatology, Hue University of Medicine and Pharmacy, Hue University, Vietnam

**Email:** [dangminhhuy@hueuni.edu.vn](mailto:dangminhhuy@hueuni.edu.vn)

**Phone:** +84935757093

**ORCID:** 0000-0002-6910-9333

# Supplementary Materials


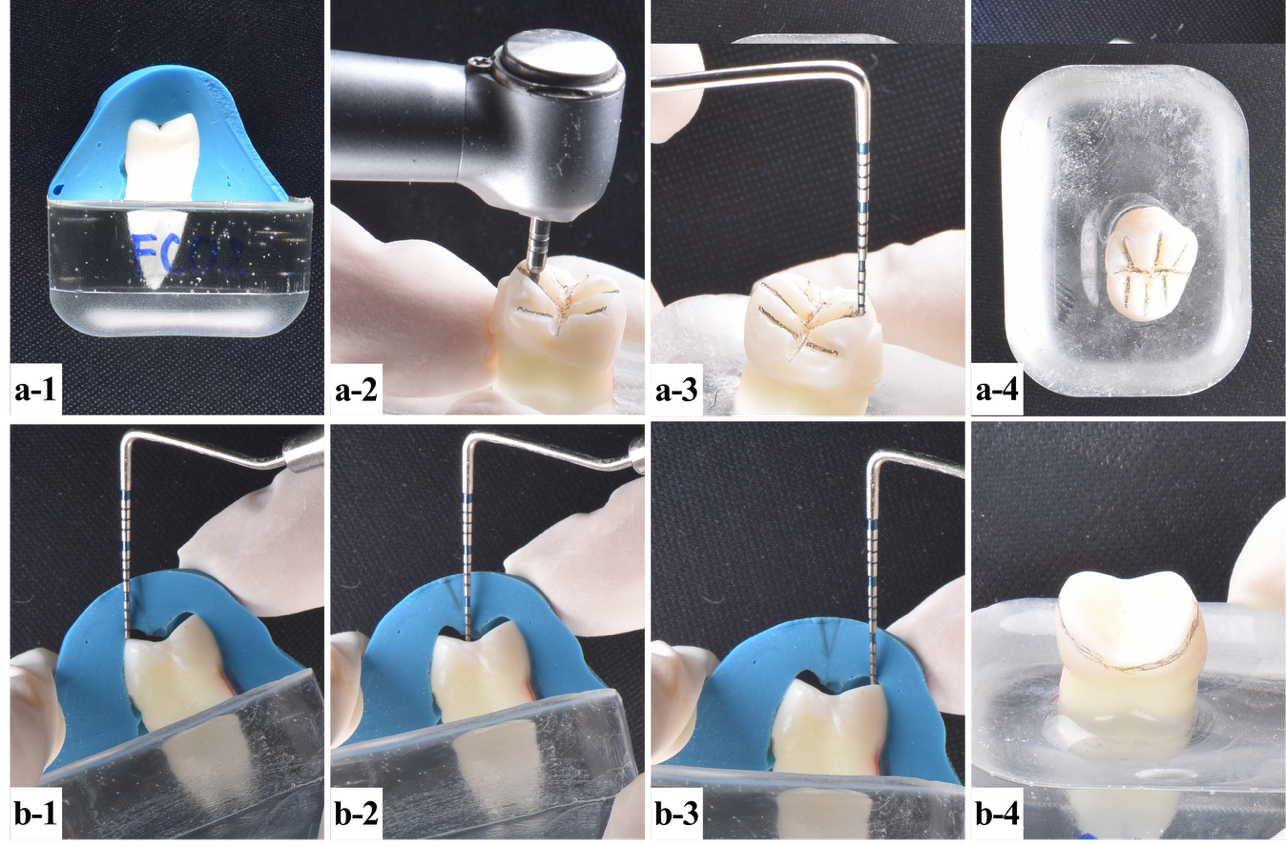


**Figure S1**. Occlusal reduction of 1-1.2 mm to replicate the tooth wear process

a-1. Silicone index; a-2. Making 1.0 mm-depth cuts to guide the uniform occlusal reduction using Komet bur PrepMarker FG DM10 314.009; a-3. Verifying groove depth using a periodontal probe; a-4. Reducing the occlusal surface by joining the depth cut grooves using Komet bur 855D.314.016 and 8370.314.030; b1-4. Verifying the occlusal reduction using the silicone index


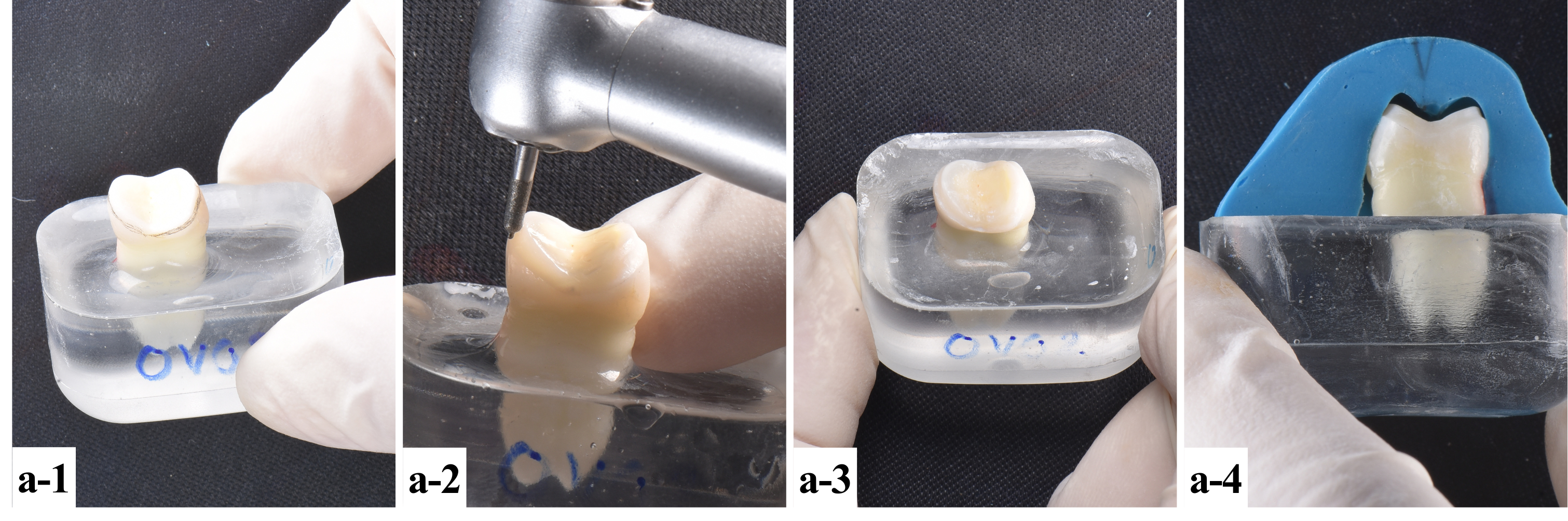


**Figure S2 (a1-4).** Overlay preparation

a1,2. Creating a 0.5-mm chamfer finish line using Komet bur 8849P.314.016; a3,4. Verifying of the preparation using the silicone index


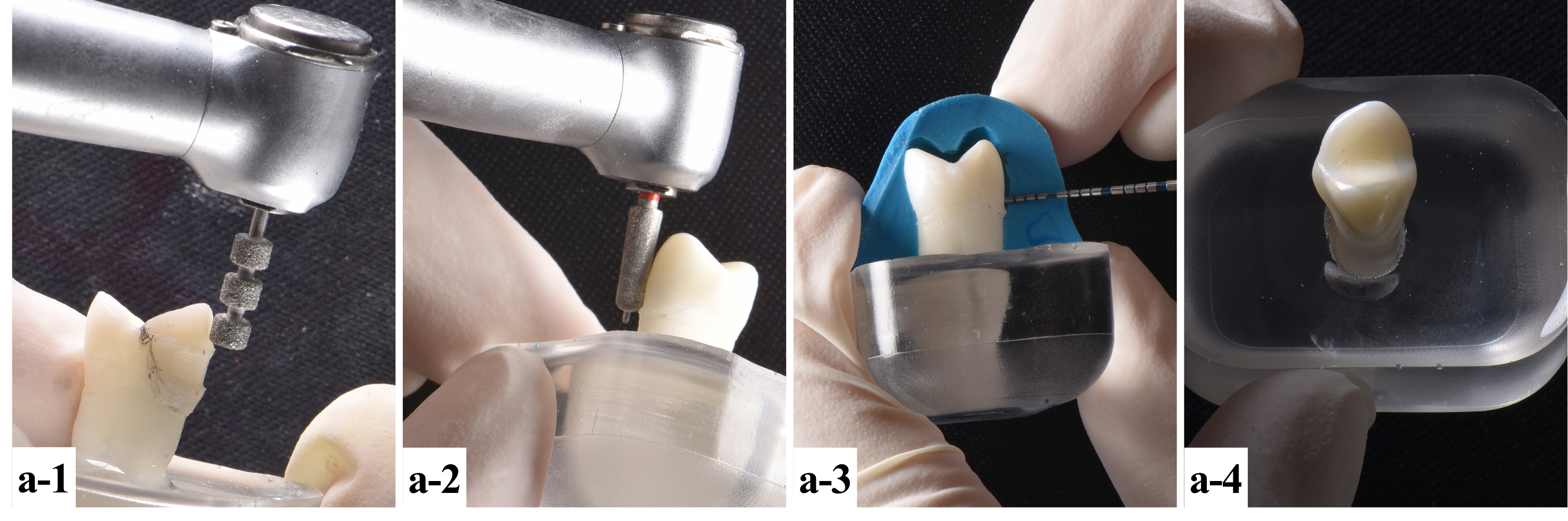


**Figure S3 (a1-4, b1-4).** Veneerlay preparation

a1,2. Making 0.8 mm-depth cuts to guide the uniform buccal reduction using NTI bur 834L-027M-FG then creating a 0.8mm chamfer finish line using NTI bur 998-021SC-FG; a3,4. Verifying the preparation using the silicone index


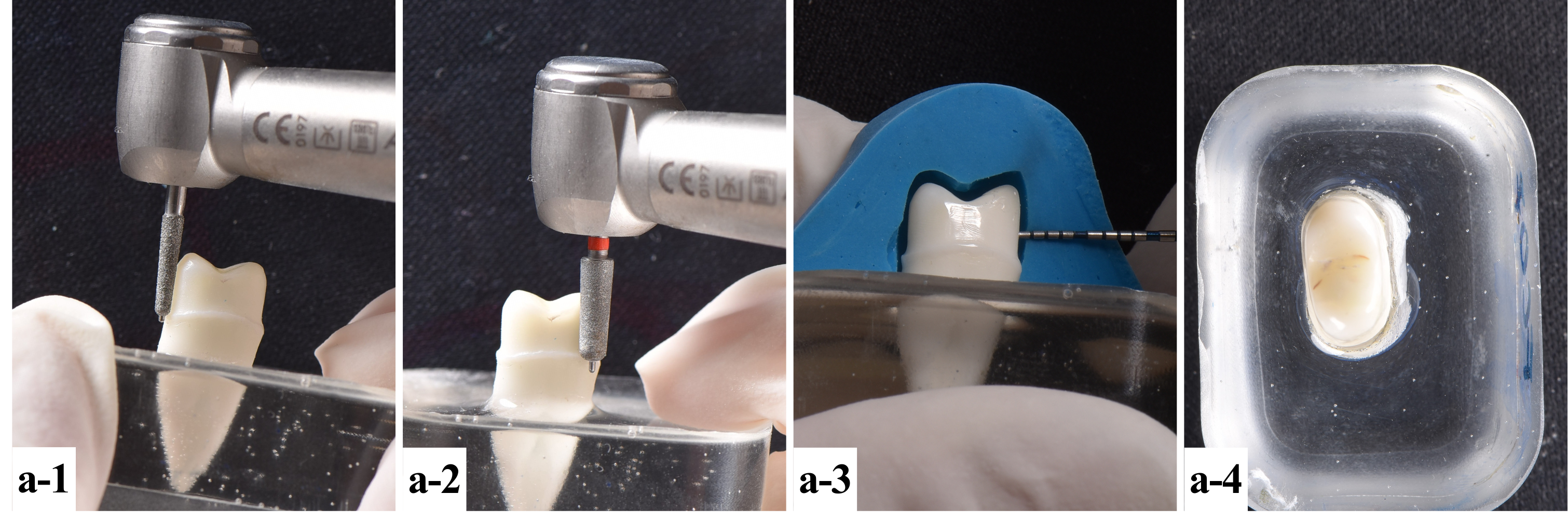


**Figure S4 (a1-4).** Full crown preparation

a1,2. Making 0.8 mm-depth cuts to guide the uniform circumferential reduction using NTI bur 834L-027M-FG and then creating 0,8mm chamfer finish line using NTI bur 998-021SC-FG; a3,4. Verifying of the preparation using the silicone index


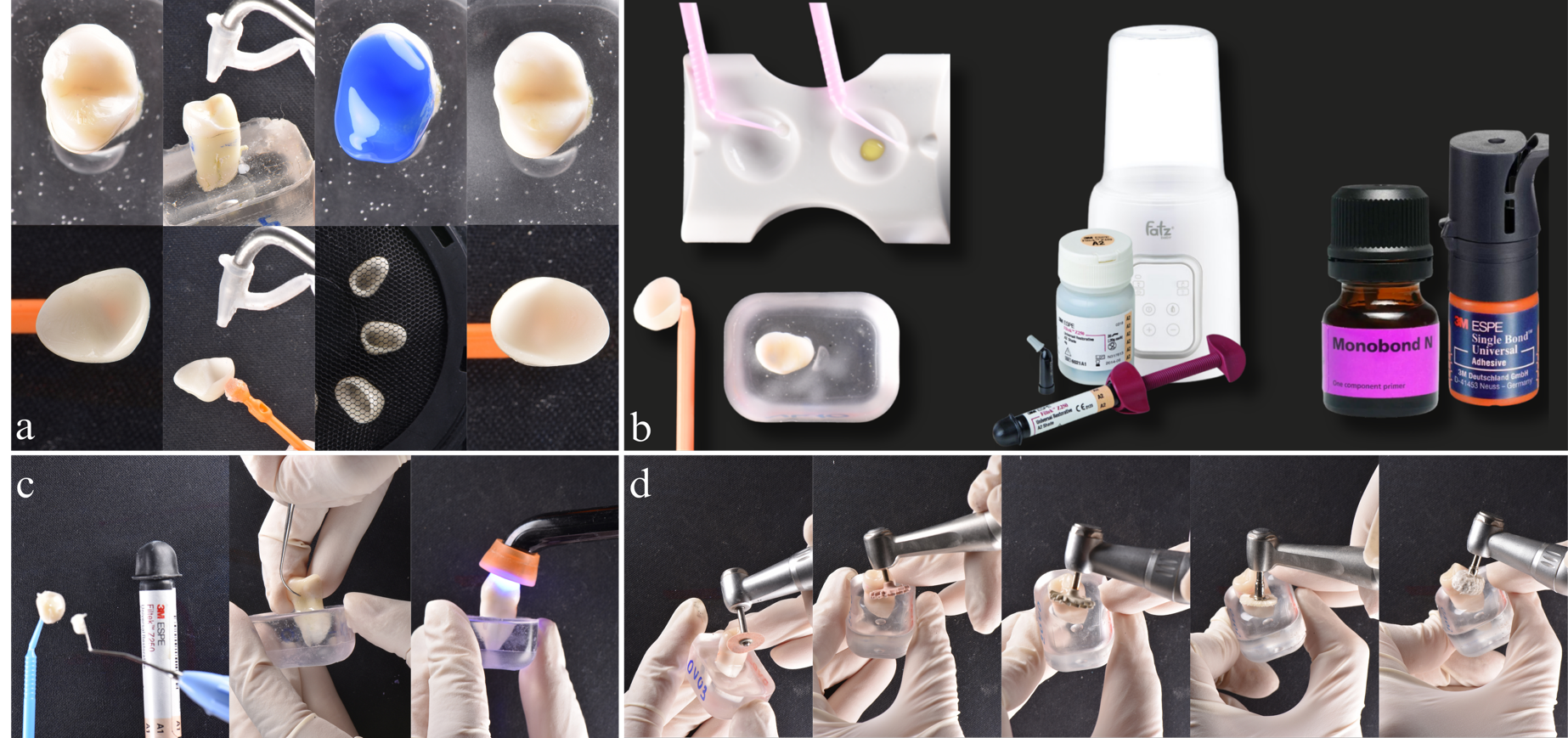


**Figure S5 (a, b, c, d).** Adhesive cementation

(a), (b) Tooth and restoration conditioning; (c) Cementation; (d) Finishing and polishing


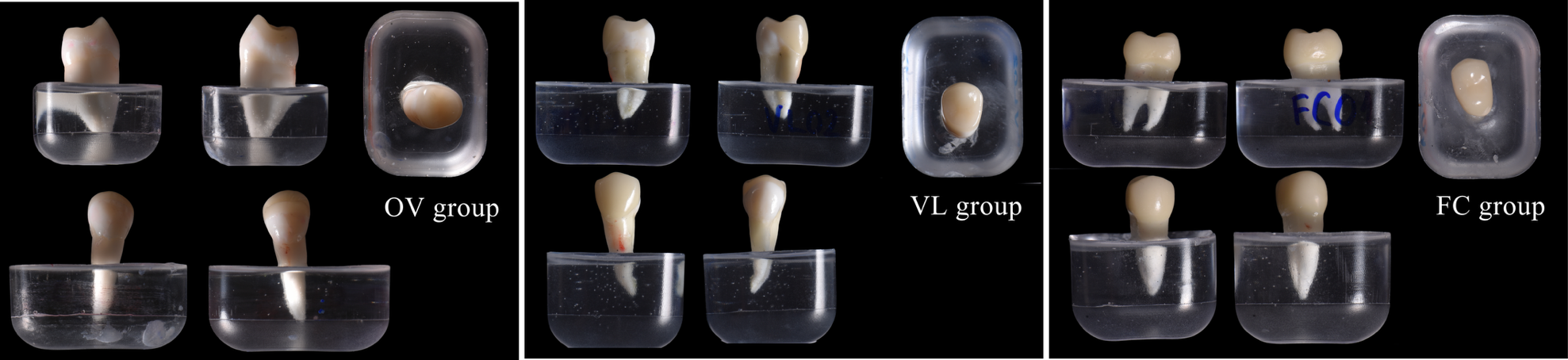


**Figure S6.** Restored groups


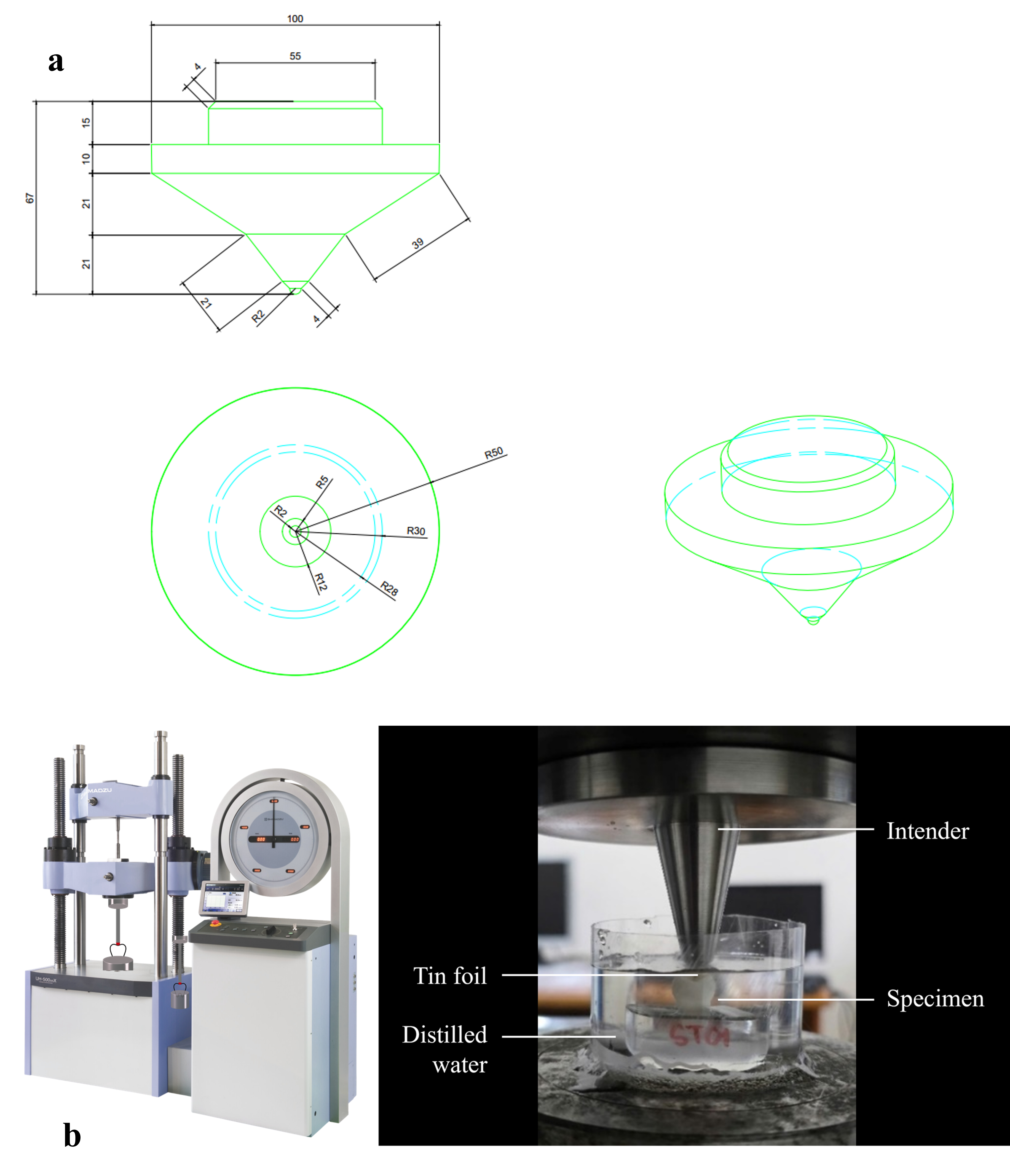


**Figure S7 (a, b).** (a) Intender design and (b) experimental set-up for the in vitro compressive load test using a universal testing machine (Shimadzu UH-500kNXh)


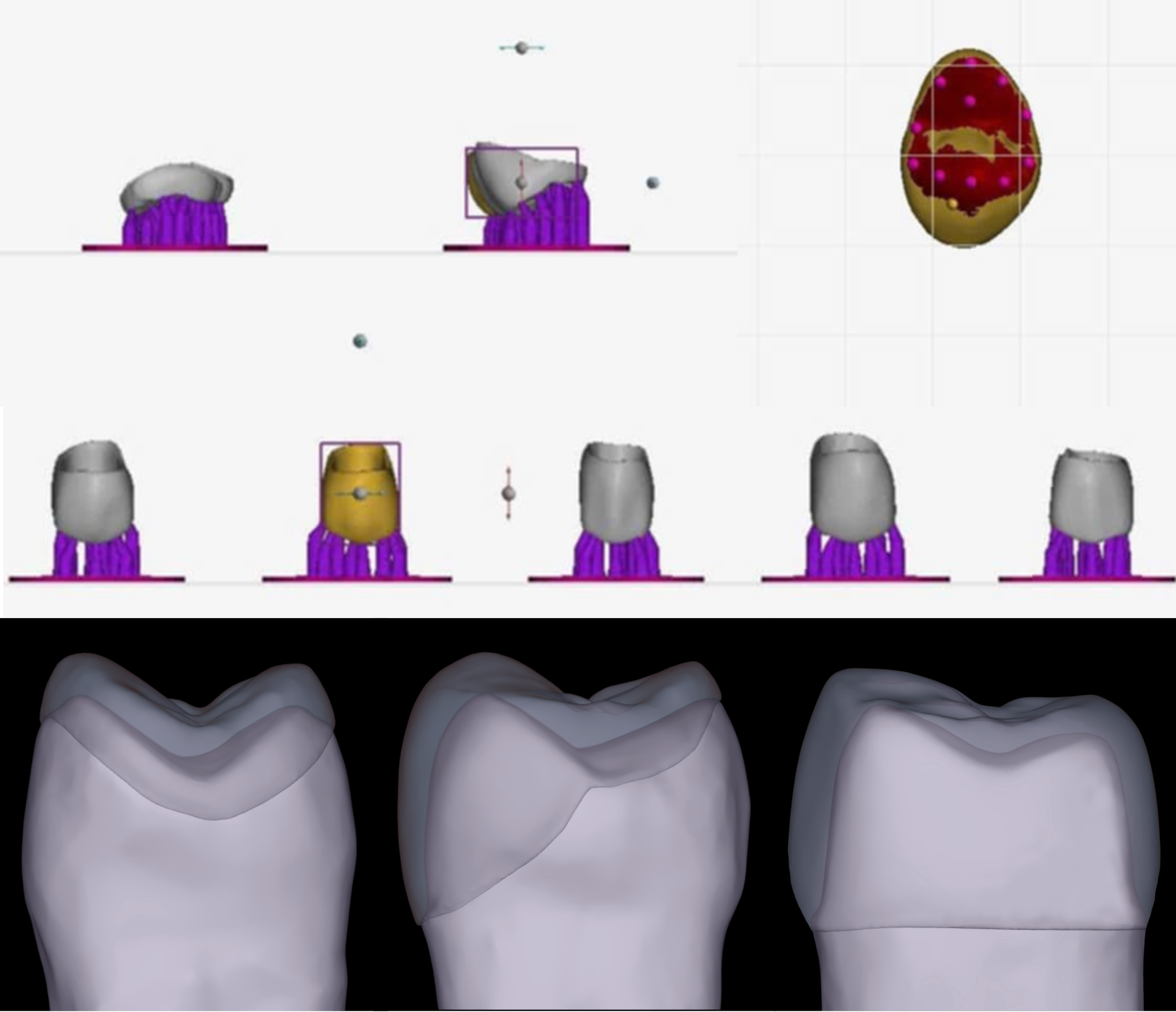


**Figure S8.** Restoration design and positioning of support tips
